# Enhancement of bacteriolysis of Shuffled phage PhiX174 gene E

**DOI:** 10.1186/1743-422X-8-206

**Published:** 2011-05-06

**Authors:** Shen-ye Yu, Wei Peng, Wei Si, Lu Yin, Si-guo Liu, Hui-fang Liu, Hai-ling Zhao, Chun-lai Wang, Yue-hong Chang, Yue-zhi Lin

**Affiliations:** 1 State Key Laboratory of Veterinary Biotechnology, Harbin Veterinary Research Institute of Chinese Academy of Agricultural Science, Harbin 150001, China

## Abstract

Bacterial ghosts that are generated using the regulated PhiX174 lysis gene E offer a new avenue for the study of inactivated vaccines. Here, we constructed a library of mutant gene E using a gene-shuffling technique. After screening and recombination with the prokaryotic non-fusion expression vector pBV220, two lysis plasmids were selected. Among which, a novel mutant E gene (named mE), consisting of a 74-bp non-encoding sequence at 5'-end and a 201-bp gene ΔE, significantly increased the lysis effect on prokaryotic *Escherichia coli *and *Salmonella enteritidis*. Moreover, lysis efficiency, as measured by the OD_600 _value, reached 1.0 (10^9 ^CFU), avoiding the bottleneck problem observed with other bacterial lysis procedures, which results in a low concentration of bacteria in suspension, and consequent low production of bacterial ghosts. Our results may provide a promising avenue for the development of bacterial ghost vaccines.

## Introduction

The lysis gene E of coliphage PhiX174 encodes a 91-amino acid (aa) residue protein of approximately 10 kDa that mediates inhibition of peptidoglycan biosynthesis of Gram-negative bacteria membrane[1]. This facilitates lysis from changes in osmotic pressure, leaving an empty bacterial body lacking cytoplasm and nucleic acids, which is called a bacterial ghost (BG) [2]. BGs retain an intact bacterial surface structure and integrated antigen proteins, and can be generated without harsh physical or chemical inactivation methods. BGs can be used directly as vaccines, as they exhibit good immunogenicity and provide effective inducible immunoprotection. Furthermore, inserting the exogenous antigen protein into the outer, inner, or periplasmic membranesis is relatively simple, and genetic engineering can be used to construct recombinant multivalent BG vaccines. Thus, BGs have been widely applied in the development of new vaccines [3-5].

In a previous study, we constructed a lysis plasmid pBV-E from the PhiX174 lysis gene E and the plasmid pBV220. Upon transformation of *E. coli *strain DH5α, lysis efficiency was 98%. Using parameters from previous studies, the optical density (OD) value at 600 nm of the bacterial suspension was maintained at 0.4 (10^6 ^CFU) by adding chloramphenicol (50 μg/mL) to inhibit bacterial proliferation [6]. Nonetheless, low lysis efficiency and ghost production have been the key technical obstacles that have prevented the large-scale production and industrialization of BG vaccines.

To address these technical problems, the current study focused on the function of the bacteriophage PhiX174 lysis gene E. Mutations were introduced by gene shuffling, and after screening and recombination with the pBV220 vector, novel mutants of lysis E gene (named mEs) were selected and the lysis plasmid pBV-mEs were characterized. Plasmids pBV-mEs may be a useful tool for producing BGs of Gram-negative bacteria such as *E. coli *and *Salmonella enteriditis*.

## Materials and methods

### Plasmids and bacterial strains

**Figure 1:**
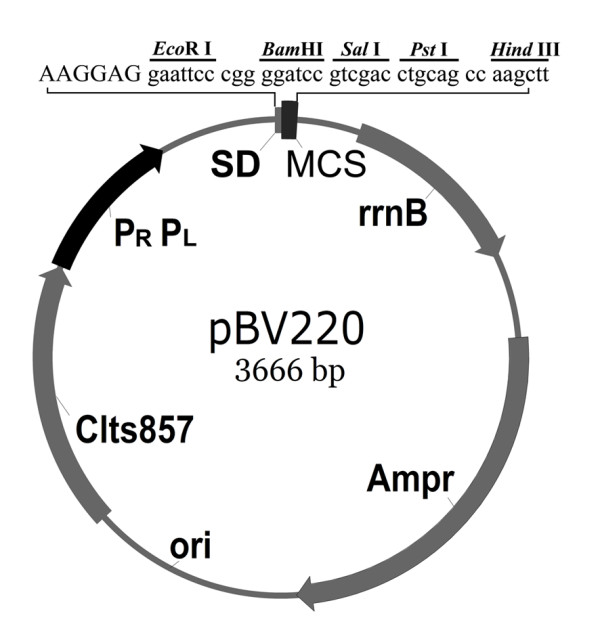
**Physical map of prokaryotic non-fusion expression vector pBV220**. Ampr, ampicillin resistance gene; ori, replication origin; cIts857, restraining gene of lambda bacteriophage adapted to heat induced expression; P_R_P_L_, tandem promoters of lambda bacteriophage for the high level expression; SD, Shine-Dalgarno sequence; rrnB, ribosome rrnB gene providing translation stop signal; and *Eco*RI and *Bam*HI, restriction enzyme sites to insert mutant lysis E gene into vector.

Plasmid pBV220 was a kind gift from Professor Gui-hong Zhang of South China Agricultural University. It is a prokaryotic non-fusion expression vector with multiple cloning sites (MCS) adjacent with Shine-Dalgarno (SD) sequence, and a high-copy number plasmid with a gene for ampicillin resistance and a λPL/PR-cI857 temperature-sensitive system that can express target gene inserted in MCS at 42°C (Figure 1). *E. coli *strain JL09 and *S. enteriditis *strain DH09 were isolated and identified in our lab. Bacteriophage PhiX174 was purchased from NEB (Beverly, MA). *E. coli *strain DH5α was maintained in our laboratory.

### Cloning of bacteriophage PhiX174 lysis gene E

Primers were designed according to the sequence of PhiX174 lysis gene E [7] in Genbank and synthesized by Invitrogen Inc. (Shanghai). Upstream primer LysisE-U: 5'-AGG GAA TTC ATG GTA CGC TGG ACT TTG TGG-3'(*Eco*R I restriction site was underlined), downstream primer LysisE-L: 5'-AGG GGA TCC GAG CTC TCA CTC CTT CCG-3'(*Bam*H I restriction site was underline). Gene E was amplified using PhiX174 double-stranded DNA as template, in a 50 μL reaction with amplification cycle parameters: 5 min at 95°C; 30 cycles of 30 s at 94°C, 30 s at 59°C, 1 min at 72°C, 5 min at 4°C. PCR products were resolved on 0.9% agarose gels, and the 300-bp gene E fragment was extracted with a gel extraction kit (Shanghai Watson Biotech Co., China).

### DNA shuffling in lysis gene E

DNA shuffling was carried out according to Evans [8] with minor modifications. The purified gene E was digested with DNase I and fragments of 10-50 bp were gel-purified and amplified by primerless PCR. Reaction parameters were: 45 cycles of 94°C for 30 s, 40°C for 30 s, and 72°C for 30 s. PCR products were analyzed on a 2% agarose gel and used as PCR templates using LysisE-U and LysisE-L as primers, with parameters: 95°C for 5 min; 30 cycles of 94°C for 30 s, 59°C for 30 s, 72°C for 30 s, and extension at 72°C for 5 min. Products were resolved on 0.9% agarose gels and extracted as above.

### Construction, screening, and transformation of clinical strains and lysis plasmid library efficiency comparison

Purified *Eco*R I- and *Bam*H I-digested fragments of mutant gene E were inserted into pBV220 and transformed into DH5α competent cells, which were spread on LB plates containing ampicillin. Single colonies were picked and screened by colony PCR with primers LysisE-U and LysisE-L. Positive clones were cultured at 37°C and sent to Invitrogen Inc. (Shanghai) for sequencing. Expression of lysis gene E or mutants of E was induced at 42°C. After bacteriolysis, the bacterial suspension was diluted to an appropriate concentration and spread on LB containing ampicillin. After 37°C for 12 h, colonies were counted and the results expressed as CFU/ml. The lysis rate was calculated using the following formula: lysis rate = (1- CFU after induction/CFU before induction) × 100%. Plasmids with high lysis rates were selected and sequenced, and those with the highest lysis rate were electroporated into the clinically isolated bacterial strains *E. coli *JL09 and *S. enteriditis *DH09. Analysis, and calculation and comparison of lysis rates used the protocol above. The corresponding bacterial strain harboring pBV220 was used as control.

### Scanning electron microscopy (SEM) and transmission electron microscopy (TEM)

Sample preparation for SEM (model, JSM-6510LV, Japan) was by centrifugation of a induced bacterial suspension at 3000 × *g *for 10 min, before fixing with 2.5% glutaraldehyde (pH 7.2) for 1.5 h at 4°C. Samples were washed three times for 10 min with 0.01 M phosphate-buffered saline (PBS). Dehydration steps were 10 min each in 50%, 70%, 80%, and 90% ethanol, a 1:1 mixture of 100% ethanol and tert-butanol, and pure tert-butanol. After lyophilization for approximately 4 h, samples were placed on the microscope platform, coated with a layer of heavy metal and examined.

Sample preparation for TEM (microscope model, JEM 1200 EX, Japan) was as above, except fixing was for 2-3 h. Samples were washed as above, and fixed with 2% osmic acid for 1.5 h, followed by three washings of 10 min each with 0.1 M PBS. Dehydration steps were 10 min each in 50%, 70%, 80%, 90%, and 100% ethanol. Samples were immersed in 1:1, 1:2, or 1:3 mixtures of SPI-Pon 812 resin (emicron) and acetone, before immersing overnight in 100% 812 resin. Resin-soaked sample blocks were polymerized at 70°C for 48 h, and samples sectioned and stained with uranyl acetate and lead citrate, before microscopy.

## Results

### PCR amplification of lysis gene E and DNA shuffling

**Figure 2:**
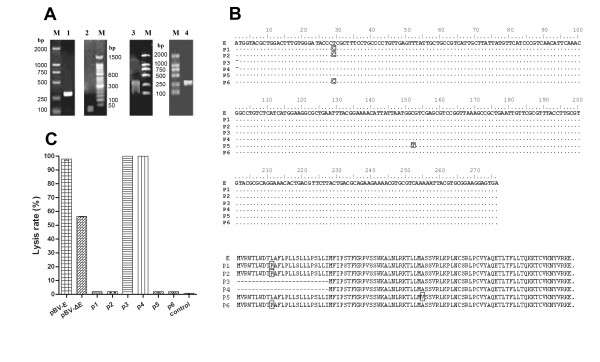
**DNA shuffling of gene E (A), nucleotide and amino acid sequences(B) and lysis efficiency comparisons of two high-efficiency lysis plasmids and four non-lytic plasmids (C)**. A1: 276 bp lysis gene E amplified from bacteriophage PhiX174. A2: Electrophoresis of the DNA fragments generated from DNase I digestion of the lysis gene E fragment. A3: Primerless PCR products. A4: Primer PCR products. B: Sequence comparison of the recombinant plasmids. Mutant sites were boxed; deletion sites were signed with strigula. C: lysis efficiency comparisons of the recombinant plasmids. pBV-E, pBV-ΔE: lysis gene E and ΔE was inserted into *Eco*R I- and *Bam*H I sites of pBV220, respectively; control: pBV220

The size of the PCR-amplified lysis gene E fragment was 276 bp, as expected (Figure 2A1). The purified fragment was digested with DNase I, and the digested fragments ran as a smear (Figure 2A2). Fragments of 10-50 bp were gel-purified and amplified by primerless PCR, and appeared as diffuse bands of 100-300 bp on a 1% agarose gel (Figure 2A3). Primerless PCR products were used as a template for primed PCR amplification, yielding a single 276-bp band on a 1% agarose gel. This band was the shuffled, mutated gene E (Figure 2A4).

### Shuffled gene E lysis plasmids, and screening of high lysis efficiency plasmids

Gel-purified mutants fragments were digested with *Eco*R I and *Bam *H I and inserted into the vector pBV220, and transformed into *E. coli *DH5α. Selection on LB containing ampicillin generated a lysis plasmid library of gene E mutants.

After large-scale screening, six stable strains were obtained. Lysis plasmids No. 3 (p3) and No. 4 (p4) had the highest lysis rates, at 99.999%. No lysis was observed in control group pBV220. Sequencing and comparison indicated that p3 and p4 share 100% identity with each other and share 98% identity with pBV-E and other four non-lysis plasmids (Figure 2B). A single nucleotide deletion was found at the 5'-end of the lysis gene E sequence in mE3 and mE4, which converts the original wild-type E gene into a 74-bp non-coding region and a 201-bp gene ΔE. Then ΔE was amplified and inserted into *Eco*R I- and *Bam*H I sites, the plasmid pBV-ΔE had lysis rate of 56.427%.(Figure 2C) Thus, this 74-bp region is likely to play an important role in lysis enhancement. We further investigated plasmid p4 (named pBV-mE), which had the highest lysis rates.

### Comparison of the pBV-mE transformed E. coli lysis curve at different OD values

**Figure 3:**
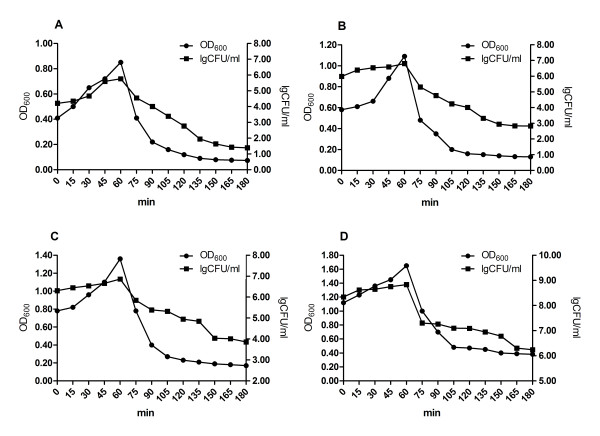
**The lysis curve of *E. coli *DH5α cells containing pBV-Me**. The lysis curve of DH5α containing pBV-mE at (A) OD_600 _= 0.4, (B) OD_600 _= 0.6, (C) OD_600 _= 0.8, (D) OD_600 _= 1.0

The results of lysis kinetics experiments showed that pBV-mE had very high lysis efficiency against DH5α cells. Effective lysis occurred at all tested OD values (0.4, 0.6, 0.8 and 1.0), and progressed rapidly, with lysis completed within 2 h (Figure 3).

### Comparison of lysis efficiencies of clinically isolated bacteria transformed with pBV-mE

**Figure 4:**
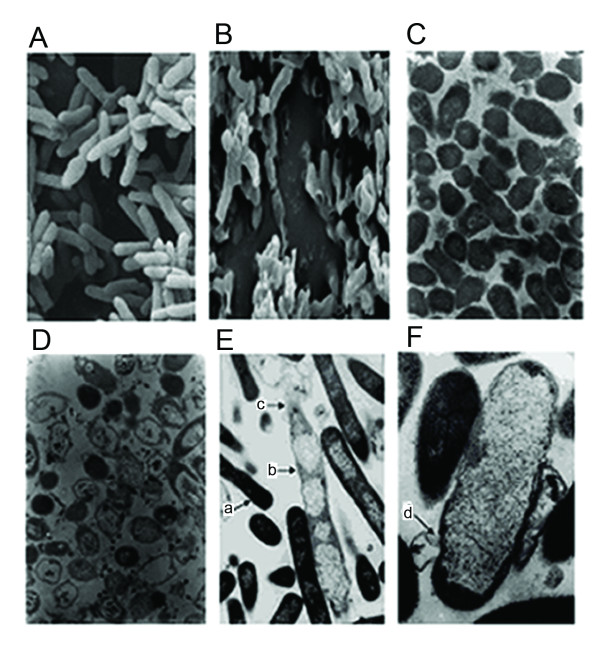
**Electron microscopy analysis of *E. coli *and *S. enteriditis *before and after lysis**. A: Morphology of wild-type *E. coli *under scanning electron microscopy: intact and healthy. B: Morphology of *E. coli *after lysis under scanning electron microscopy: shrinkage observed. C: Morphology of wild-type *S. enteriditis *under transmission electron microscopy: even electron density. D: Morphology of *S. enteriditis *ghosts after lysis under transmission electron microscopy: uneven electron density, vacuolar. E, F: Observation of the ejection of the intracellular contents from *S. enteriditis *under transmission electron microscopy. a. *S. enteriditis *before lysis (12,000 × magnification). b. *S. enteriditis *after lysis, bacterial ghost (12,000 ×). c. Lysis tunnels on ghosts (12,000 ×). d. Lysis tunnels on ghosts (30,000 ×)

Plasmid pBV-mE had the same lysis effect on both *E. coli *and *S. enteriditis*. Comparison of SEM and TEM photographs of the cell surface of *E. coli *and *S. enteriditis *before and after lysis showed that BGs retained the basic cell morphology of the bacterial cells, but displayed obvious surface wrinkles from the loss of intracellular contents. Arrow c in Figure 4E, and arrow d in Figure 4F indicate lysis tunnels in the *S. enteriditis *BGs, located mainly at the cell poles. Ejection of the intracellular contents from the lysis tunnels can be seen, consistent with a previous report [9], although the BGs in our study exhibited relatively complete outer membranes with little change in morphology.

## Discussion

Genetic inactivation of Gram-negative bacteria by inducing a regulated bacteriophage PhiX174 lysis gene E provides a new avenue for inactivated vaccine research [4,10]. E-protein-mediated lysis has already been used to investigate many types of bacterial ghosts, suggesting that it may be applicable to any of the Gram-negative bacteria [3,11-16]. Moreover, bacterial ghosts can be used as vehicles for the delivery of exogenous antigens or drugs.

In this study, a 275-bp mE gene was selected from a library of mutant lysis gene E generated by DNA shuffling. The mutant contained a 74-bp non-encoding sequence and a 201-bp lysis gene ΔE. A lysis plasmid containing the mE gene driven by dual pR and pL promoters significantly increased lysis efficiency over the 95% efficiency in the same *E. coli *strain with previously reported constructs with a single pR or pL promoter [17]. The lysis efficiency of the plasmids was not affected by bacterial concentration. Bacteria with an OD_600 _of 1.0 (10^9^cfu) are sufficiently concentrated for vaccination, and the observed 99.999% lysis efficiency of pBV-mE was higher than another lysis plasmid, pBV-E (98%) that we previously generated [6]. The new lysis plasmid eliminates the lysis requirement that the OD of the bacterial culture be between 0.4 and 0.6, which has been a key technical problem for large-scale vaccine production. Therefore, the current data indicate a promising avenue for BG vaccine research.

While both *E. coli *and *Salmonella *showed good lysability, the lysis efficiency of protein mE was significantly higher than lysis protein E in both bacteria. Previous studies showed that the N-terminal 51 aa of PhiX174 lysis protein E are necessary for lysis activity [18] and the lysis activity localized to the N-terminal 29 aa [19]. Another study suggested that the activity was associated with the N-terminal 35 aa, including a hydrophobic area (a potential transmembrane region) that is essential for the lysis function of protein E [20]. The C-terminus of protein E is important for conformational stability and regulation of lysis functions [21]. It was proposed that, at an early stage of bacteriolysis, a conformational isomerism probably occurs at proline residue 21 (P21) in the first transmembrane α-helix of protein E. Mutations of P21 to alanine, glycine, serine, or valine result in an unstable protein, and thus affect E protein levels *in vivo *and the loss of lysis function. The initiation of cell division, rather than any specific functions of the septotome has been shown to play a pivotal role in protein E-mediated Gram-negative bacteriolysis [22].

It is interesting that our results differ from those of previous studies. For plasmid p1 and p6 with observed enhancement of lysis effect, single deletion of A from the 5'-end of E gene converted the first 75-bp encoding sequence to 74-bp non-encoding region, which resulted in the loss of first 25 aa in wild-type E protein. Considering the length of the mE protein is sufficient for crossing the membrane, we propose that lysis activity is not located only within the first 29 aa of protein E, but rather that sequences downstream of aa 25 also have lysis activity. However, so far as now, the detailed mechanism of E-mediated lysis is still obscure, let alone the mE protein. Therefore, we would work on the assumption as follows.

**Figure 5:**
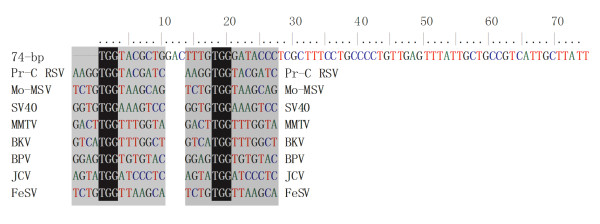
**Comparison of 74-bp sequence with other enhancers**. Two possible enhancer core sequences in gray shade are similar to the reported core enhancer sequence "GTGGT(A) T(A) T(A)G". Pr-C RSV, Prague C Rous Sarcoma Virus; Mo-MSV, Moloney Murine Sarcoma Virus; SV40, Simian Vaculating Virus-40; MMTV, Mouse Mammary Tumor Virus; BKV, BK Virus; BPV, Bovine Papilloma Virus; JCV, JC Virus; FeSV, Feline Sarcoma Virus

It should be pointed out that, after sequence comparison, we found the similarity of this 74-bp sequence with many well-known viral enhancers (Figure 5). Moreover, the first five bases (TGGTA) of the 74-bp is only one nucleotide different from the minus-35 region of the RpoN (alternate sigma factor) consensus (TGGCA). It is also only one nucleotide different from the minus-35 sequence of the strong promoter of the recA gene (TGATA). Therefore, one possible explanation for this enhancement is that the 74-bp sequence dramatically improves the transcription efficiency of pBV-mE, increases production of the ΔE protein and provides excess protein molecule for lysis. Another explanation is the 74-bp sequence may positively regulate the replication of lysis plasmids pBV-mEs. All lysis plasmids constructed with mutant E genes (pBV-mEs) had copy numbers higher than that of pBV-E (data not shown). Lin et al. reported that copy numbers of pUC family plasmids are much higher than other plasmids with a pMB1 or colE1 origin. This is due to a single point mutation in the pUC plasmids that can cause a temperature-dependent alteration in the secondary structure of RNA II[23].

In conclusion, the results from our study could indicate a potentially new avenue to solve two key problems in BG research and vaccine production: low bacterial concentration and low BG production. More work is needed to confirm the above assumptions about mE gene. Identification and characterization of 74-bp enhancer or enhance-like or promoter-like element is carrying out.

## Competing interests

The authors declare that they have no competing interests.

## Authors' contributions

SYY did laboratory testing, analyzed the test results, wrote and edited the manuscript. WP and WS did laboratory testing and co-wrote the manuscript. LY, HFL, HLZ, CLW, YHC and YZL did laboratory testing. SGL is the leader of the study group and organized the overall project and helped edit the manuscript. All the authors read and approve the final manuscript.
